# Supplementary material for: Whole-Genome Analysis Illustrates Global Clonal Population Structure of the Ubiquitous Dermatophyte Pathogen Trichophyton rubrum
Source: Genetics. 2018 Feb 20;208(4):1657–69. doi: 10.1534/genetics.117.300573 (PMC5887155; doi:10.1534/genetics.117.300573)
Supplement: Supplementary file 1 [file 1657FigureS1.pdf]

T. interdigitale H6	CCTGCGGAAGGATCATTAACGCGCAGGCCGGAGGCTGGCCCCCCACGATAGGGCCAAACGTCCGTCAGGGGTGAGCAGATGTG
T. interdigitale MR816	CCTGCGGAAGGATCATTAGCGCGCAGGCCGGAGGCTGGCCCCCCACGATAGGGCCAAACGTCCGTCAGGGGTGAGCAGATGTG
T. interdigitale AF168124	CCTGCGGAAGGATCATTAGCGCGCAGGCCGGAGGCTGGCCCCCCACGATAGGGCCAAACGTCCGTCAGGGGTGAGCAGATGTG
T. interdigitale AY062119	CCTGCGGAAGGATCATTAACGCGCAGGCCGGAGGCTGGCCCCCCACGATAGGGCCAAACGTCCGTCAGGGGTGAGCAGATGTG

T. interdigitale H6	CGCCGGCCGTACCGCCCCATTCTTGTCTACATTACTCGGTTGCCTCGGCGGGCCGCGCTCTCCCAGGAGAGCCGTTCGGCGAG
T. interdigitale MR816	CGCCGGCCGTACCGCCCCATTCTTGTCTACATTACTCGGTTGCCTCGGCGGGCCGCGCTCTCCCAGGAGAGCCGTTCGGCGAG
T. interdigitale AF168124	CGCCGGCCGTACCGCCCCATTCTTGTCTACATTACTCGGTTGCCTCGGCGGGCCGCGCTCTCCCAGGAGAGCCGTTCGGCGAG
T. interdigitale AY062119	CGCCGGCCGTACCGCCCCATTCTTGTCTACCTTACTCGGTTGCCTCGGCGGGCCGCGCTCTCCCAGGAGAGCCGTTCGGCGAG

Figure S1. ITS sequence variation in *T. interdigitale*. Aligned ITS sequence is shown for four isolates, including the two for which whole genomes were sequenced (H6 and MR816) and two previously characterized isolates (AF168124 and AY062119). Isolate AY062119 has been re-classified as *T. mentagrophytes*. Variant sites are highlighted.
