# Supplementary material for: Whole-Genome Analysis Illustrates Global Clonal Population Structure of the Ubiquitous Dermatophyte Pathogen Trichophyton rubrum
Source: Genetics. 2018 Feb 20;208(4):1657–69. doi: 10.1534/genetics.117.300573 (PMC5887155; doi:10.1534/genetics.117.300573)
Supplement: Supplementary file 2 [file 1657FigureS2.pdf]

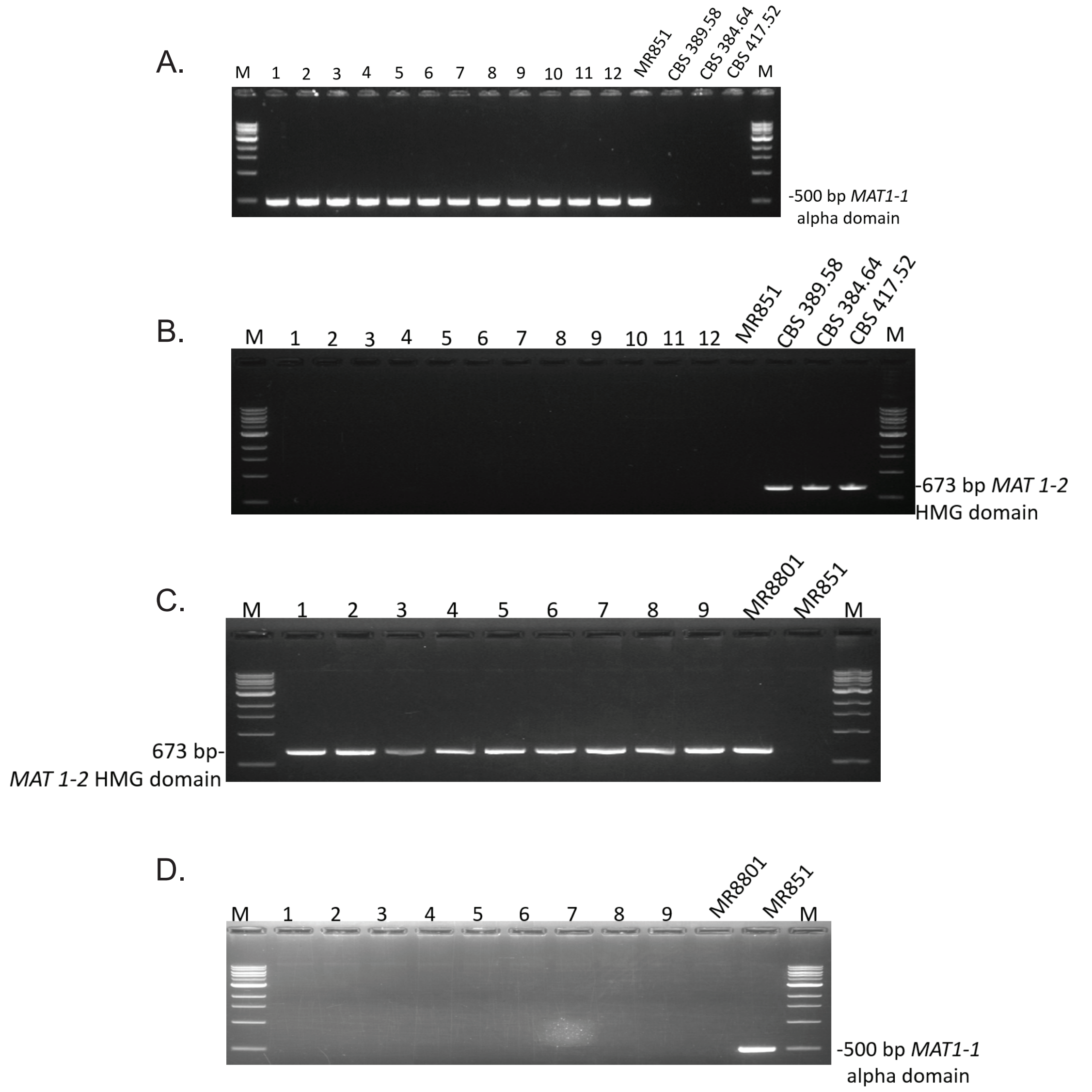

Figure S2. Detection of MAT1-1 in *T. rubrum* and MAT1-2 in *T. interdigitale* by PCR. A. PCR based determination of the MAT1-1 alpha domain of *T. rubrum* strains: *T. rubrum* MR 851, *T. megninii* CBS 389.58, *T. megninii* CBS 384.64, and *T. megninii* CBS 417.52. The alpha domain MAT1-1 gene was identified from samples = 1-12 and MR 851 *T. rubrum*, alpha domain MAT1-1 gene was not identified from *T. megninii* CBS 389.58, *T. megninii* CBS 384.64, or *T. megninii* CBS 417.52. M: DNA ladder. B. PCR based determination of MAT 1-2 HMG domain of *T. rubrum*: *T. rubrum* MR851, *T. megninii* CBS 389.58, *T. megninii* CBS 384.64, and *T. megninii* CBS 417.52. The HMG domain MAT1-2 gene was identified from *T. megninii* CBS 389.58, *T. megninii* CBS 384.64, *T. megninii* CBS 417.52, and was not identified from *T. rubrum* isolate = 1-12 and *T. rubrum* MR 851, M: DNA ladder. C. PCR based determination of MAT1-2 HMG domain of *T. interdigitale*. *T. interdigitale* strains = 1-9, *T. interdigitale* MR8801 positive control, and *T. rubrum* MR851 as negative control, M; DNA ladder. D. PCR based determination of MAT 1-1 alpha domain of *T. interdigitale* strains. *T. interdigitale* strains = 1-9, *T. interdigitale* MR8801 negative control, and *T. rubrum* MR851 positive control, M; DNA ladder.
