# Supplementary material for: Whole-Genome Analysis Illustrates Global Clonal Population Structure of the Ubiquitous Dermatophyte Pathogen Trichophyton rubrum
Source: Genetics. 2018 Feb 20;208(4):1657–69. doi: 10.1534/genetics.117.300573 (PMC5887155; doi:10.1534/genetics.117.300573)
Supplement: Supplementary file 3 [file 1657FigureS3.pdf]

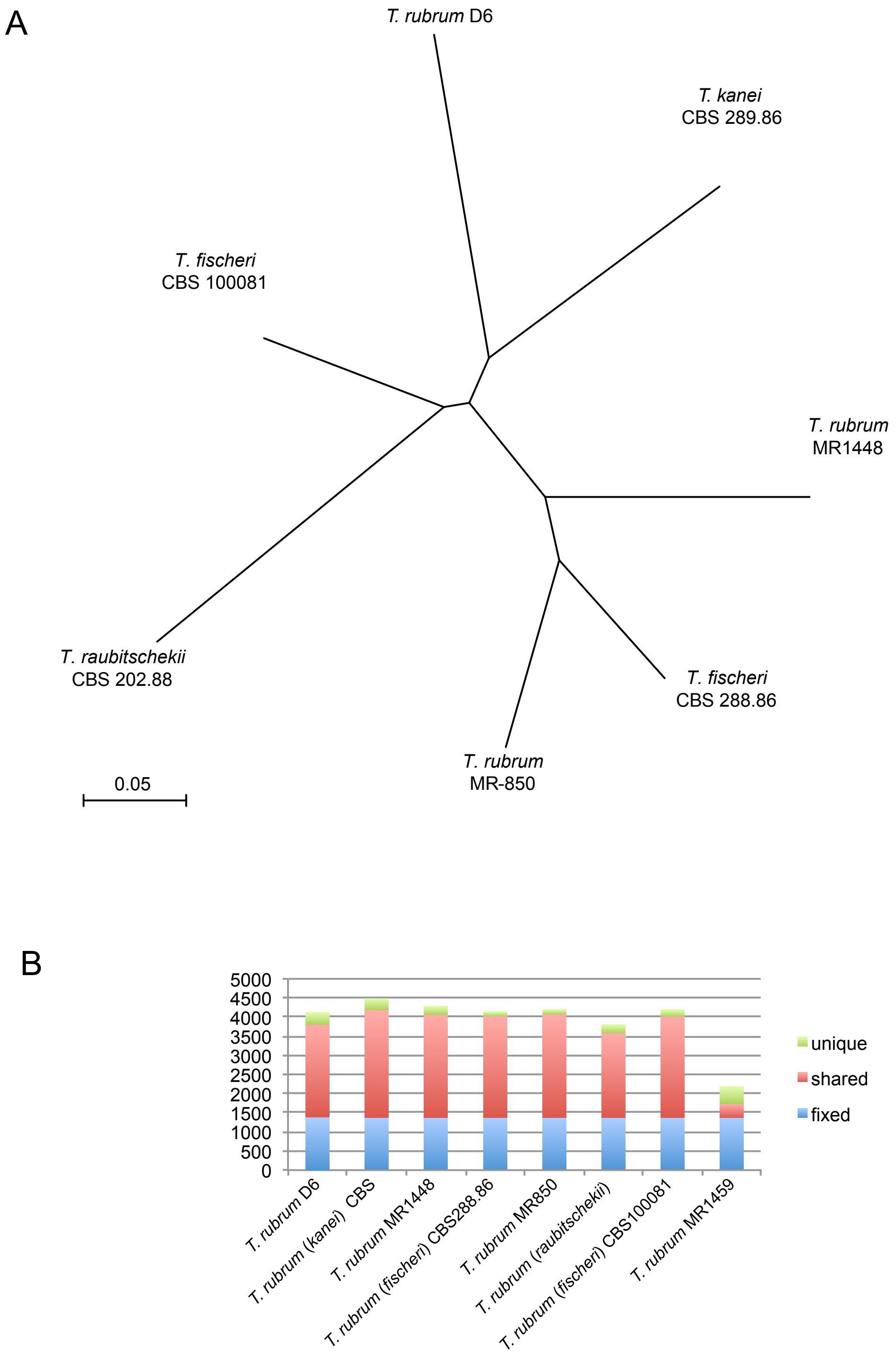

Figure S3. Phylogenetic relationship and sharing of variant sites of sequenced *T. rubrum* isolates. A. Phylogenetic relationship of *T. rubrum* isolates inferred using RAxML (Methods). B. Classification of SNP sites based on conservation across the sequenced isolates; unique: only in one isolate; shared: in two to seven isolates; fixed: in all eight isolates.
