# Supplementary material for: Whole-Genome Analysis Illustrates Global Clonal Population Structure of the Ubiquitous Dermatophyte Pathogen Trichophyton rubrum
Source: Genetics. 2018 Feb 20;208(4):1657–69. doi: 10.1534/genetics.117.300573 (PMC5887155; doi:10.1534/genetics.117.300573)
Supplement: Supplementary file 4 [file 1657FigureS4.pdf]

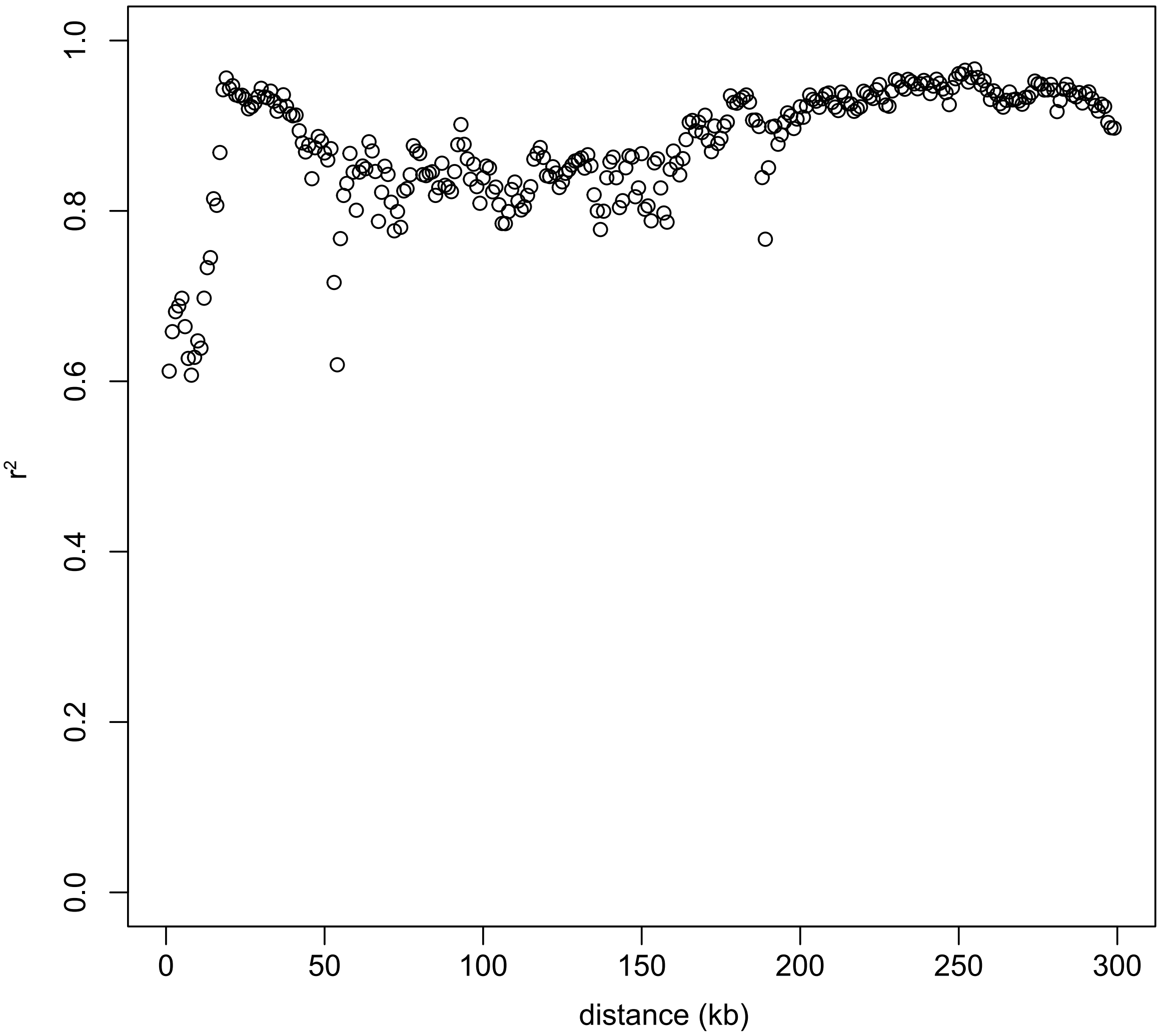

Figure S4. Lack of decay of linkage disequilibrium (LD) in *T. rubrum*. LD (r^2) was calculated for all pairs of SNPs separated by 0–300 kb and then averaged for every 1kb. LD values for each window were then calculated by averaging over all pairwise calculations in the window.
