# Supplementary material for: Whole-Genome Analysis Illustrates Global Clonal Population Structure of the Ubiquitous Dermatophyte Pathogen Trichophyton rubrum
Source: Genetics. 2018 Feb 20;208(4):1657–69. doi: 10.1534/genetics.117.300573 (PMC5887155; doi:10.1534/genetics.117.300573)
Supplement: Supplementary file 5 [file 1657FigureS5.pdf]

A.

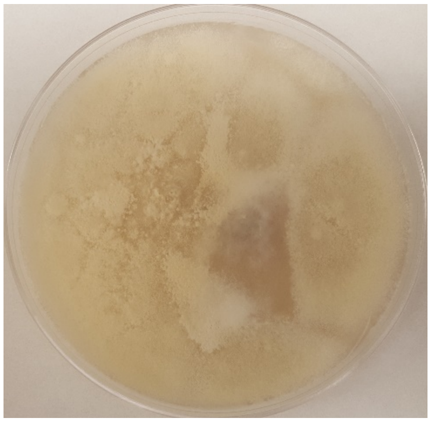

B.

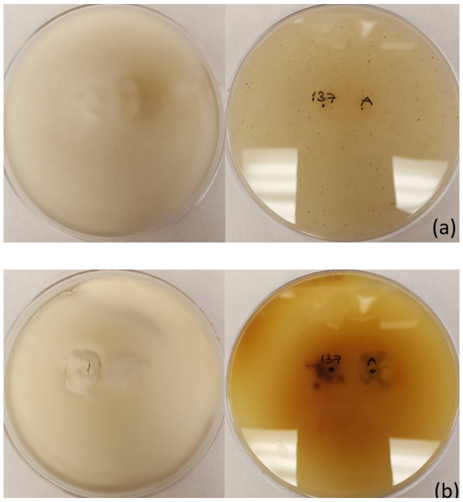

C.

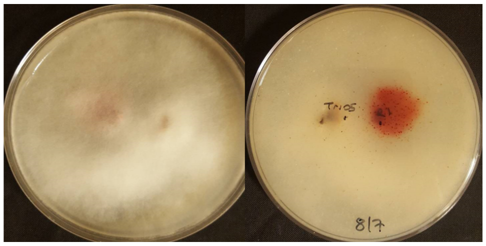

Figure S5. Mating assays. A. Mating assay plate; *T. rubrum* and *T. interdigitale* on E medium. B. Mating assay plate*; T. rubrum* and *A. simii* (a) E medium, (b) Takashio medium eight weeks. C*. T. rubrum* and *T. megninii* on E medium eight weeks.
