# Supplementary material for: Whole-Genome Analysis Illustrates Global Clonal Population Structure of the Ubiquitous Dermatophyte Pathogen Trichophyton rubrum
Source: Genetics. 2018 Feb 20;208(4):1657–69. doi: 10.1534/genetics.117.300573 (PMC5887155; doi:10.1534/genetics.117.300573)
Supplement: Supplementary file 6 [file 1657FigureS6.pdf]

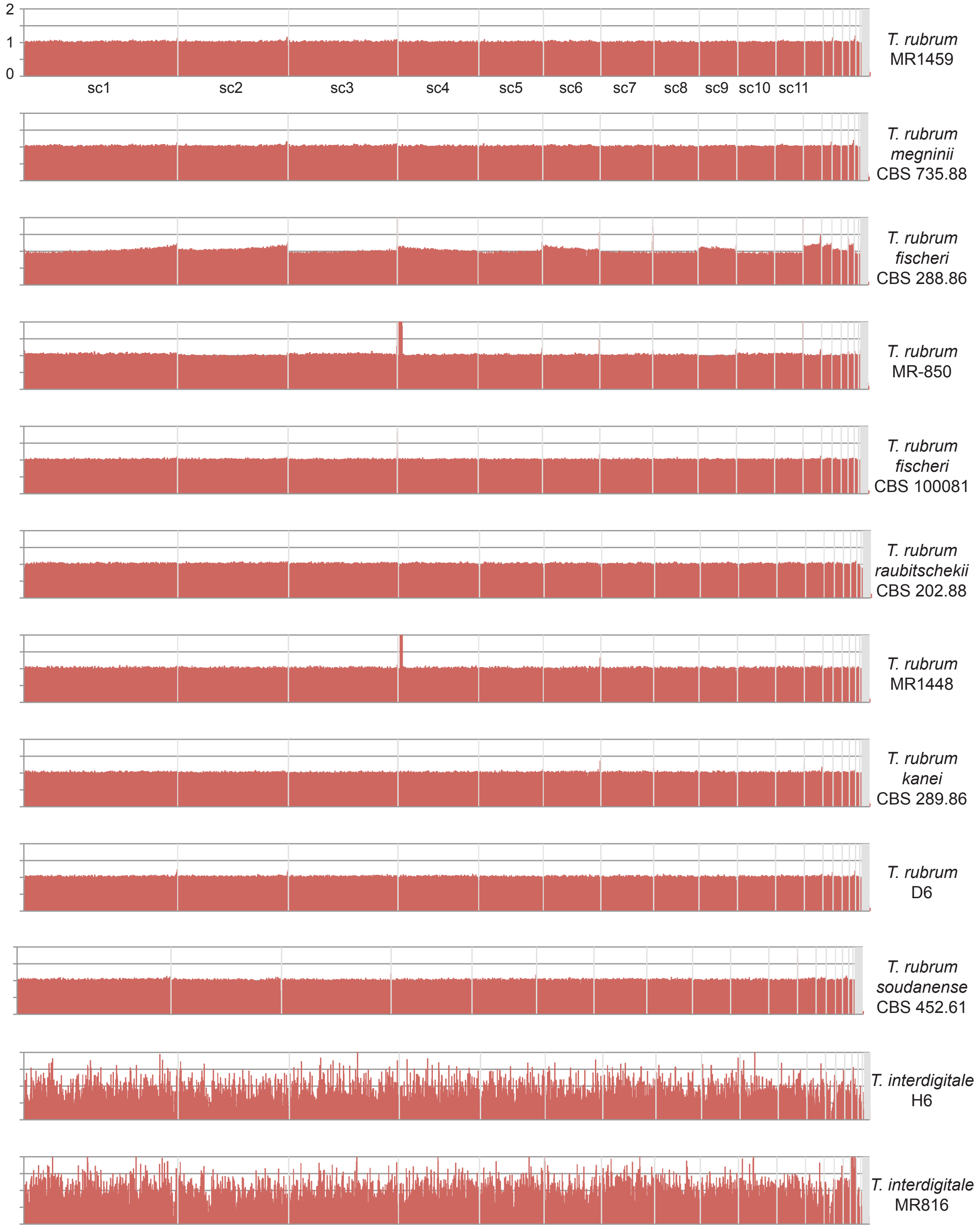

Figure S6. Read depth of sequenced isolates. Reads from each isolate were aligned to the *T. rubrum* reference genome and normalized read depth computed for 5kb windows. Read depth is even across the reference genome for most isolates, with small regions of higher depth detected in some isolates.
