# Supplementary material for: Whole-Genome Analysis Illustrates Global Clonal Population Structure of the Ubiquitous Dermatophyte Pathogen Trichophyton rubrum
Source: Genetics. 2018 Feb 20;208(4):1657–69. doi: 10.1534/genetics.117.300573 (PMC5887155; doi:10.1534/genetics.117.300573)
Supplement: Supplementary file 7 [file 1657FigureS7.pdf]

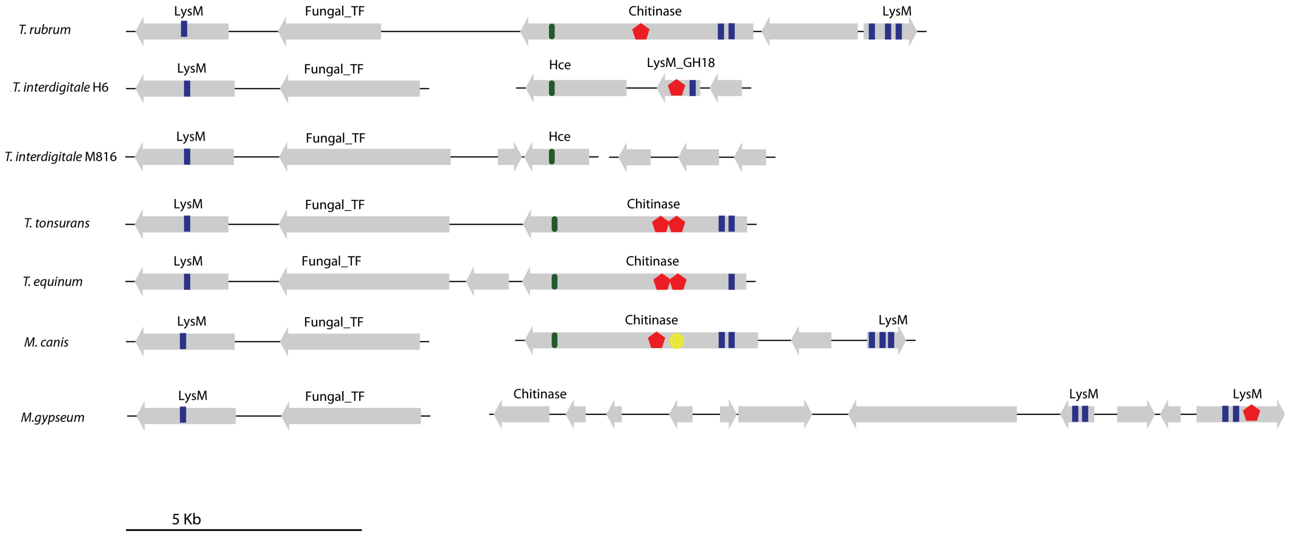

Figure S7. Variation in LysM-Hce gene cluster across sequenced Dermatophytes. In *T. rubrum*, the LysM-Hce gene is closely linked to two other LysM genes; this organization is most similar to that found in *M. canis*, although these genes are located on two different scaffolds.
